# Roles of Non-coding RNAs in Central Nervous System Axon Regeneration

**DOI:** 10.3389/fnins.2021.630633

**Published:** 2021-02-01

**Authors:** Pei Li, Yuanyuan Jia, Wenbo Tang, Qingjun Cui, Ming Liu, Jingjing Jiang

**Affiliations:** Department of Anesthesiology, Shengjing Hospital of China Medical University, Shenyang, China

**Keywords:** non-coding RNA, nerve regeneration, central nervous system, optic nerve injury, spinal cord injury

## Abstract

Axons in the central nervous system often fail to regenerate after injury due to the limited intrinsic regeneration ability of the central nervous system (CNS) and complex extracellular inhibitory factors. Therefore, it is of vital importance to have a better understanding of potential methods to promote the regeneration capability of injured nerves. Evidence has shown that non-coding RNAs play an essential role in nerve regeneration, especially long non-coding RNA (lncRNA), microRNA (miRNA), and circular RNA (circRNA). In this review, we profile their separate roles in axon regeneration after CNS injuries, such as spinal cord injury (SCI) and optic nerve injury. In addition, we also reveal the interactive networks among non-coding RNAs.

## Introduction

Central nerve injuries are commonly seen in clinical practice. They can severely affect quality of life and may be fatal. Unfortunately, there is still no known cure for these kinds of injuries because of their complex pathophysiology. Damaged axons in the central nervous system (CNS) rarely regenerate, mainly due to the inhibitory environment around the damaged nerves and a lack of intrinsic neuronal growth ability (Goncalves et al., 2015). In the search for a cure for CNS injuries, researchers have made a leap in progress in terms of the mechanisms of neuronal regeneration. Evidence shows that multiple transcriptional regulatory pathways can promote the capacity of neurite regrowth (Sun et al., 2011; Apara et al., 2017; Wang Z. et al., 2018). Non-coding RNAs, which used to be regarded as transcription noise, are now proven to be important transcriptional and post-transcriptional regulators in the development of the nervous system and neurological diseases (Wu et al., 2013). In recent years, the functions of non-coding RNAs in axon regeneration in the peripheral nervous system (PNS) have continuously been revealed, which has provided clues about CNS axon regeneration. Researchers have found that multiple non-coding RNAs are differentially expressed after CNS injuries, such as spinal cord injury (SCI) and optic nerve injury, which implies that non-coding RNAs may have the potential to become brand new biomarkers and targeted therapies for axon regeneration in the CNS. Therefore, in this review, we first profile the extrinsic and intrinsic mechanisms for nerve regeneration; then, we give a brief introduction on long non-coding RNA (lncRNA), microRNA (miRNA), and circular RNA (circRNA), which are widely associated with axon regeneration. Finally, we focus on some typical non-coding RNAs to demonstrate the separate and interactive functions of non-coding RNAs in nerve regeneration. Last but not least, we also suggest potential directions for future research.

### Extrinsic and Intrinsic Mechanisms for Nerve Regeneration

After nerve damage in nervous systems, successful compensation may include axonal sprouting from intact neurons or regeneration from injured axons, therefore, bringing about function recovery. Unlike the robust regrowth in the peripheral nervous system (PNS) following injury, the central nervous system (CNS) appears to have defects related to nerve regeneration (Oh et al., 2018). In mammals, this process relies both on the improvement of a permissive extracellular environment (Tom et al., 2004) and intrinsic growth ability (Goncalves et al., 2015). In the last century, Aguayo et al. revealed that axons in an injured spinal cord and brainstem could successfully regrow over long distances when the CNS glial environment was replaced by peripheral nerves (David and Aguayo, 1981), showing that the inhibitory microenvironment attributes to the failure of axon regeneration in the CNS. Over time, varieties of extrinsic factors were discovered to have a relationship with the regeneration process after CNS injury. Glial scars, myelin debris, and axonal growth inhibitors form significant impediments to axonal regeneration (Li et al., 2020). Glial scars, which serve a primary role in preventing inflammatory processes from propagating to healthy tissue (Cregg et al., 2014), also create a physical barrier in which axonal tips get trapped. Confronted with the scar tissue, the ends of regenerating axons cease to extend and turn into abnormal growth cones that can remain for years without robust regeneration (Tom et al., 2004). Moreover, demyelination occurs after nerve damage, which severely impairs axon survival as the myelinating cells of the CNS, oligodendrocytes, are lost through injury. Under permissive conditions, they can be replaced by oligodendrocyte progenitor cells and differentiate into mature oligodendrocytes. However, myelin debris inhibits the differentiation process, so it is of vital importance to clear the myelin debris following demyelination (Church et al., 2017). Apart from that, a variety of neurite growth inhibitory factors were found to strongly suppress the sprouting and regeneration of injured neurites after CNS injury. In addition to myelin-associated glycoprotein (MAG), Nogo-A, and oligodendrocyte-myelin glycoprotein (OMgp), which are expressed by the myelin sheath in injured axons (Gribble and Scott, 2002), chondroitin sulfate proteoglycans (CSPGs), released by hypertrophic astrocytes at the lesioned site, present a potent barrier to axon regeneration (Yiu and He, 2006). Although we can remove molecules that restrict regrowth in the extracellular environment, the effect of regeneration is insufficient (Holmes, 2017). As a result, more attention has been focused on how to promote the inherent growing ability of the lesioned axons. As is known, gene transcription and protein translation play a key role in this course. Researchers have found that a series of genes take part in intrinsic regulation following CNS injury, including Pten, Klf4/9, Socs3, B-RAF, c-Myc, GSK3, and Lin28 (Moore et al., 2009; Luo and Park, 2012; Saijilafu et al., 2013; O’Donovan et al., 2014; Wang X.W. et al., 2018; Zhang et al., 2018; Ma et al., 2019). Moreover, many regeneration-associated signaling pathways, such as cAMP/PKA,DLK/JNK, are continually being uncovered (Neumann et al., 2002; Larhammar et al., 2017). However, with the development of transcriptomics, mounting studies suggest that non-coding RNAs, especially lncRNA, miRNA, and circRNA, exert their function as regulators in multiple biological programs. More importantly, they are widely found to be differentially expressed in the central nervous system after an injury (Yao et al., 2015), which indicates that they have the potential to become clinical strategies for axon regeneration after nerve injury.

### Classification and Biogenesis of Non-coding RNAs

There are two types of non-coding RNAs. The first type, constitutive non-coding RNAs, include transfer RNA (tRNA), ribosomal RNA (rRNA), small nuclear RNA (snRNA), small nucleolar RNA (snoRNA), and small cytoplasmic RNA (scRNA). Their abundance is constant, and they play an important role in maintaining the normal physiological function of species. The second type, regulatory non-coding RNAs, include lncRNA, miRNA, circRNA, siRNA, and piRNA. Their abundance changes with the external environment and cell characteristics, which play an important role in regulating gene expression. In this review, we focus on three members of the non-coding RNA family–lncRNA, microRNA, and circRNA–and describe their functions in nerve regeneration as well as their combined effects.

**FIGURE 1:**
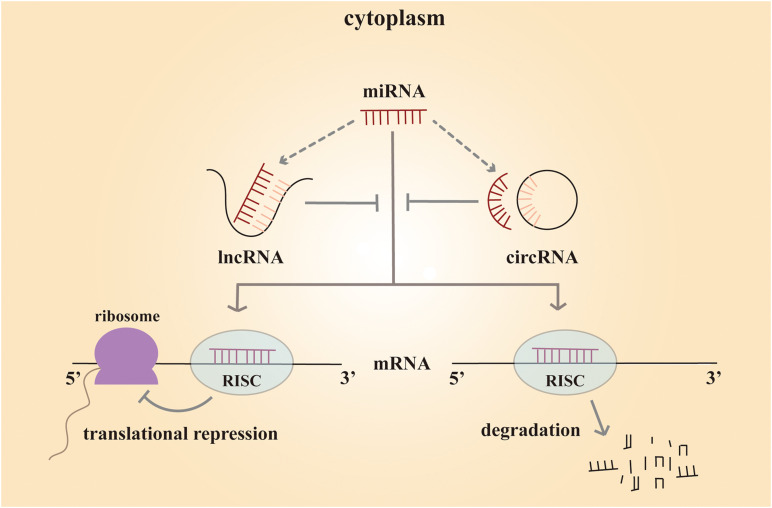
Mechanism scheme of ceRNA network. The mature miRNA regulates the gene expression by forming a RISC (RNA-induced silencing complex) and binding to the complementary sequences in their 3′ untranslated regions of the target mRNA. As a result, the miRNA silences the gene through mRNA degradation or transcriptional repression. Both lncRNAs and circRNAs are rich in binding sites for miRNAs and serve as miRNA sponges in cells. They compete with mRNA for opportunities to bind with miRNAs. In this way, lncRNAs and circRNAs remove the miRNA’s inhibition to the target mRNA, and the expression of the mRNA increases as a consequence. This mechanism is called competing endogenous RNA (ceRNA).

lncRNAs are a cluster of non-coding RNAs of more than 200 nucleotides in length (Paraskevopoulou and Hatzigeorgiou, 2016). These non-coding RNAs are poorly conserved and play a significant role at different levels, including chromatin remodeling, transcriptional control, and post-transcriptional processing. Such variable regulatory patterns suggest that lncRNA might have both complex biological and pathological functions. microRNAs are 20∼25 nucleotides in length, and they often regulate gene expression at the post-transcription level. Mature miRNA functions by binding to the complementary sequences in the 3′ untranslated regions of the target messenger RNA (mRNA). As a result, the miRNA silences the gene through mRNA degradation or transcriptional repression (Butz et al., 2012). circRNAs are a novel class of non-coding RNAs discovered in 2012. Unlike traditional linear non-coding RNAs with 5′ cap and 3′ tail structures, circRNAs are characterized by a closed-loop structure, which is more stable (Wu et al., 2019). These RNA molecules are rich in binding sites for miRNAs and serve as miRNA sponges in cells (Hansen et al., 2013). By binding to different miRNAs, they remove miRNA inhibition to the target mRNA, and expression of the mRNA increases as a result. This type of RNA is called competing endogenous RNA (ceRNA). By working with miRNAs, circRNAs play a crucial role in biological and pathological processes (Figure 1).

### Non-coding RNAs in Central Nerve Regeneration

#### Spinal Cord Injury

SCI is a devastating traumatic event that damages sensory, motor, or autonomic functions and, therefore, affects quality of life (Fehlings et al., 2017). Every year, up to a million people experience spinal cord injury. More than half of them suffer from complete injury, losing function completely in the area controlled by the lesioned nerves. The others only recover part of their body function (Holmes, 2017). The management of acute SCI requires long-term, high-level care resources, and it brings substantial financial burden at both individual and societal levels (Fehlings et al., 2017). As a result, quite a lot of attention has been paid to the mechanisms of SCI to seek better repairing strategies.

As far as we are concerned, the pathophysiology of SCI temporally changes, consisting of two injury phases. The primary mechanical injury phase damages the axons, blood vessels, and cell membranes. Within hours or days after the injury, it enters the secondary injury phase with ischemia, edema, inflammation, and vascular changes (Rowland et al., 2008; Rouanet et al., 2017). The pathophysiological changes of SCI are so complicated that a curable strategy remains a big challenge. Despite the fact that a few repair studies are making some progress in animal models, to date, clinical translation to patients has not been convincing (Dietz, 2016). Mounting evidence has shown that multiple non-coding RNAs are differentially expressed after injury. Changes in lncRNA, miRNA, and circRNA are so evident that much attention has been focused on them (Chen Y. et al., 2016; Zhou et al., 2018; Wu et al., 2019). The non-coding RNAs are strongly connected to the various pathophysiological processes of SCI, such as inflammation, apoptosis, axon regeneration, oxidative stress, and astrocyte proliferation and activation (Li et al., 2016; Gu et al., 2017; Xia et al., 2018; Yao and Yu, 2019). Moreover, they have been found to play a potential role in SCI complications, including neuropathic pain (Wang F. et al., 2019). Here, we review recent studies on non-coding RNAs and their mechanisms of axon regeneration after spinal cord injury.

Accumulated evidence suggests that miRNAs are strongly related to the regulation process after SCI. Bioinformatics analysis demonstrated expression changes of a cluster of miRNAs *in vivo*, concluding that miRNAs may take part in promoting self-repair in SCI (Liu et al., 2016; Wang W. et al., 2019). In previous studies, micro-125b showed a significant decrease in expression level after SCI, and overexpression of microRNA-125b promotes axon regeneration following spinal cord injury by regulating the JAK/STAT pathway. Furthermore, micro-125b displays a positive neuronal protective effect by reducing apoptosis and inflammatory response in neurons (Dai et al., 2018). microRNA-155 deletion has been shown to reduce inflammatory signaling in macrophages, which enhances axon regeneration dynamics. In addition, microRNA-155 knockout neurons in adults also show independent enhanced axon growth (Gaudet et al., 2016). Moreover, some researchers point out that, regulated by TGF-β1, microRNA-21-5p mediates the expression of fibrosis-related genes. microRNA-21-5p knockdown attenuates the formation of fibrotic scars, which are thought to be the main physical obstacle for axonal regeneration following SCI (Wang W. et al., 2018). These results demonstrate that microRNAs might be a novel target for axon regeneration in SCI patients.

lncRNAs have been shown to have significant effects on the development of the brain (Goff et al., 2015), nervous systems (Zhang et al., 2019), and a variety of neurological diseases, such as brain tumors (Voce et al., 2019), neurodegenerative diseases (Chanda et al., 2018; Cao et al., 2019), and ischemic strokes (Hu et al., 2020). Moreover, lncRNAs also serve as a crucial regulator in SCI, affecting its initiation and progression. Several studies have demonstrated that they mostly perform functions via cell autophagy and apoptosis (Salah et al., 2020). However, some evidence tells us that lncRNAs probably also play a role in regulating axonal regrowth following SCI. Some previous studies have suggested that lncRNAs are of vital importance to neurite outgrowth, which is an early step in neuronal regeneration. It has been shown that lncRNA Malat1 plays a crucial role in neurite outgrowth in N2a cells by activating the MAPK/ERK signaling pathway. The MAPK family is thought to take part in a variety of biological processes such as development and apoptosis. Furthermore, the data show that elevated expression of lncRNA Malat1 promotes the average neurite length and thus enhances neuronal differentiation (Chen L. et al., 2016). Moreover, lncRNA-Map2k4 was found to have a high expression level and decline in SCI pathology. Data shows that lncRNA-Map2k4 regulates neuron proliferation through the microRNA-199a/FGF1 axis. Knockdown of lncRNA-Map2k4 inhibited FGF1 expression by upregulating microRNA-199a (Lv, 2017). As is known, FGF1 is a neurotrophic factor with a potent neuroregenerative ability in the nervous system (Ding et al., 2016), so this finding may provide some new ideas for the treatment of SCI.

In recent decades, more investigations into circRNAs have taken place, and circRNAs have been found to have a variety of functions in various disorders, such as cancers, cardiovascular diseases, diabetes, and ischemic strokes (Siede et al., 2017; Bai et al., 2018; Li et al., 2019; Abbaszadeh-Goudarzi et al., 2020). A number of studies using bioinformatics analysis have revealed that circRNAs are differentially expressed in central nerve injuries. One study explored the implication of circRNA expression profiles in spinal cord injury in rats at the immediate phase. Using microarray analysis, researchers found that 1,101 circRNAs were upregulated and 897 circRNAs were downregulated in SCI rats at the immediate phase compared with sham control rats. Additionally, enrichment analysis displayed that dysregulated circRNAs were enriched in many biological processes, such as spinal cord development and synapse assembly. They were also enriched in signaling pathways related to neuronal signal transduction and inflammation, such as the MAPK signaling pathway and axon guidance (Liu et al., 2020). In another study, 150 circRNAs were differentially expressed in spinal cords from an SCI group compared with the sham-injured control group. Among these, 99 circRNAs were upregulated, and 51 were downregulated. Further analysis disclosed that circRNA_07079 and circRNA_01282, two of the dysregulated circRNAs, were associated with SCI and might play a role in the pathophysiology of SCI (Zhou et al., 2019).

#### Optic Nerve Regeneration

Like the spinal cord, the optic nerve is also a part of the CNS, which has little ability to regenerate after it has been injured. As a result, optic nerve injuries and some degenerative diseases, such as traffic trauma, ischemic injury, and glaucoma, have become leading causes of irreversible blindness worldwide. Up to now, there are still no satisfactory treatments for this type of visual impairment, which is primarily due to the complex mechanisms underlying the pathological process (Li et al., 2017). Retina ganglion cells (RGCs) make up the innermost layer of neurons in the retina, of which the axons extend out to form the optic nerve. It has been reported that the irreversible loss of vision is attributed to RGCs losing the ability to regenerate axons (Mak et al., 2020). Therefore, axon regeneration becomes critical to recovering function after optic nerve impairment. While many transcription factors and proteins have had their functions related to axon regeneration after optic nerve injury uncovered, some mechanisms still remain elusive (Park et al., 2008; Duan et al., 2015; Li et al., 2015). Subsequently, researchers found that non-coding RNAs, especially microRNAs (miRs), are differentially expressed during RGC development and affect axon regeneration.

One previous study found that the developmental decline of axon regenerative ability in RGCs is in accordance with the expression levels of the miR-17-92 family members. Their data also showed that the developmental downregulation of miR-19a, one of the family members, is related to the upregulated expression of phosphatase and tensin homolog deleted on chromosome 10 (PTEN) in RGCs. It is believed that miR-19a promotes axon regeneration in both mature rodent RGCs and human adult RGCs by suppressing PTEN, a negative regulator of the mammalian target of the rapamycin (mTOR) pathway, which controls cell growth by regulating cap-dependent protein translation initiation (Park et al., 2008). In addition, with the development of RGCs, miR-19a expression is drastically reduced, which contributes to the decline of axon regenerative capacity (Mak et al., 2020). In another study, microRNA-30b (miR-30b) was found to inhibit Semaphorin3A (Sema3A) expression in RGCs by bonding to its 3′ UTR. Sema3A is a very potent repulsive molecule and an important inhibitory factor involved in CNS repair following damage. miR-30b can promote axon regeneration by reducing the binding ability of Sema3A to NRP1/PlexA1, both of which are receptors, in order to transmit a signal of growth cone collapse. Moreover, miR-30b also reduces RGC apoptosis by inhibiting the expression of p-p38MAPK and active caspase 3 (Han et al., 2015). Some findings have demonstrated that inhibition of miR-21 attenuates excessive astrocyte activation and glial scar formation by regulating the epidermal growth factor receptor (EGFR) pathway, thereby promoting axon regeneration in RGCs and function recovery in flash visual evoked potential (F-VEP) in the rat model of optic nerve injury (Li et al., 2018). Apart from these results, miR-135s has also been proven to facilitate RGC axon regeneration after optic nerve injury in adult mice, in part by repressing Krüppel-like factor 4 (KLF4), which is a well-known intrinsic inhibitor of axon regeneration (van Battum et al., 2018). In addition, another study suggests that let7-miRNAs act downstream of Lin28a/b and serve as negative regulators of axon regeneration. Overexpression of Lin28a can induce robust and sustainable optic nerve regeneration without affecting the survival rate of RGCs (Wang W. et al., 2018). All the above highlight the potential of microRNAs to serve as a new tool in the treatment of optic nerve impairments.

Many circRNAs are expressed in different stages in the retina. One study pointed out that silencing circTulp4 would lead to a thin outer nuclear layer and defective retinal function. In addition, they found that circRNAs were dysregulated at a much earlier time point than disease onset in a retinal degeneration model (Chen et al., 2020). These results provide some clues for investigating the underlying mechanisms and potential repairing targets of CNS injuries. However, the functions of circRNAs in axon regeneration are still elusive and require further investigation.

#### Networks of Non-coding RNAs

With the development of high-throughput sequencing technology, we can obtain a lot of complete transcriptome information at one time. Through bioinformatics analysis, interestingly, researchers found that, apart from the separate functions of the non-coding RNAs in central nerve regeneration, they can also interact with each other to produce a marked effect. Recently, miRNA has been verified to be affected by the presence of miRNA sponge transcripts, the so-called competing endogenous RNAs (Hansen et al., 2013). Either lncRNA or circRNA can be miRNA sponges and thus affect the expression of target mRNAs, which is true of interactive networks among the two non-coding RNAs.

Using microarray analysis, Liu et al. (2020) constructed a circRNA-miRNA network of 10 candidate circRNAs in a spinal cord injury rat model using miRanda and found that most of the dysregulated circRNAs have a number of target miRNAs. In another study, results showed that retinal circRNAs could act as miRNA sponges. They also discovered that circTulp4 functioned as a ceRNA by acting as a sponge for miR-204-5p and miR-26a-5p to regulate retina development (Chen et al., 2020). Wang et al. found that lncRNA33755 and circRNA6370 were targets of miR-21-5p after SCI and displayed an lncRNA/circRNA-miRNA-mRNA axis (Wang W. et al., 2019), which implies the possibility of an analogous axis in central nerve regeneration. One research team suggested that lncRNA-Map2k4 was the target gene of miR-199a. Finally, they regulated FGF1 expression, which is essential to axon regeneration, as we discussed above (Lv, 2017).

## Discussion

**FIGURE 2:**
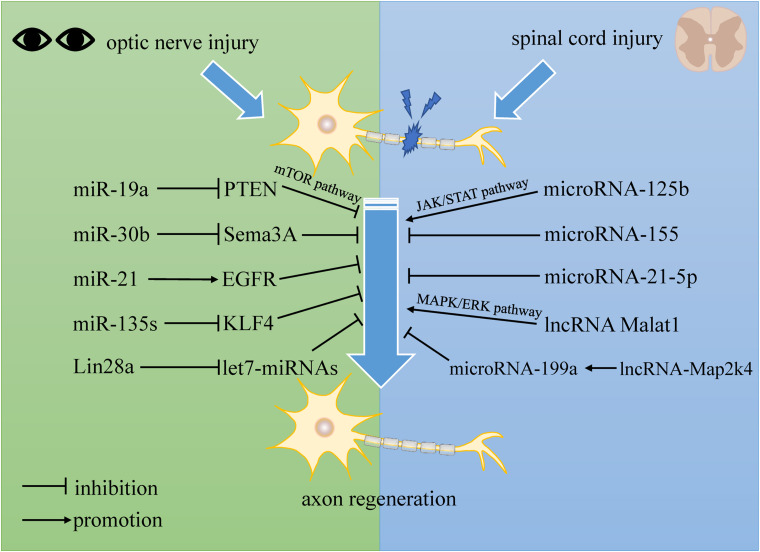
The regulation of non-coding RNAs to central nerve regeneration.

In this review, we summarized the extrinsic and intrinsic mechanisms for central nerve regeneration, especially non-coding RNAs. After central nerve injury, a quantity of non-coding RNAs perform differential expression, which implies their potential functions in repairing the nervous system. Table 1 lists recent studies with an overall profile of their roles in axon regeneration after CNS injuries, such as SCI and optic nerve injury. We not only elaborate on the separate functions of lncRNA, microRNA, and circRNA, but also reveal interactive networks among the non-coding RNAs (see Figure 2).

**TABLE 1:** Differentiated expression of non-coding RNAs in different CNS injuries.

	Injury model	*In vitro*/*in vivo*	Tissue/cell	Non-coding RNA	Expression patterns	Target	Function	References
Spinal cord injury	C5 spinal blunt contusion (mouse)	*In vivo*	Spinal cord	microRNA-125b	Upregulation	JAK/STAT pathway	Reduce apoptosis and inflammatory response in neurons	Dai et al., 2018
	Spinal cord dorsal peripheral conditioning lesion/column crush Injury (mouse)	*In vivo*	Spinal cord	microRNA-155	Downregulation	Unclear	Reduce inflammatory signaling in macrophages	Gaudet et al., 2016
	Spinal cord contusion (mouse)	*In vivo*	Epicenter spinal cord	microRNA-21-5p	Downregulation	Unclear	Attenuate the formation of fibrotic scars	Wang W. et al., 2018
	**−**	*In vitro*	Neurons-spinal cord	lncRNA-Map2k4	Upregulation	MicroRNA-199a/FGF1 axis	Regulate neuron proliferation	Xia et al., 2018
	Spinal cord contusion (rat)	*In vivo*	Spinal cord	circRNA_07079/circRNA_01282	Upregulation	miR-351-5p (predicted)	Unclear	Zhou et al., 2019
	Allen’s weight-drop model (mouse)	*In vivo*	Epicenter spinal cord	miR-21-5p	Upregulation	lncRNA33755/circRNA6370	Unclear	Wang W. et al., 2019
Optic nerve injury	Optic nerve crush (mouse and human)	*In vitro*/*in vivo*	RGC	miR-19a	Upregulation	PTEN	Control cell growth by regulating protein translation initiation	Park et al., 2008; Mak et al., 2020
	Optic nerve crush (rat)	*In vivo*	RGC	microRNA-30b	Upregulation	Sema3A/p-p38MAPK and caspase-3	Reduce growth cone collapse and RGC apoptosis	Han et al., 2015
	Optic nerve crush (rat)	*In vitro*/*in vivo*	Optic nerve/astrocyte	miR-21	Downregulation	EGFR receptor pathway	Attenuate excessive astrocyte activation and glial scar formation	Li et al., 2018
	Optic nerve crush (mouse)	*In vitro*/*in vivo*	SH-SY5Y cell/RGC/cortex/hippocampus	miR-135s	Upregulation	KLF4	Facilitate RGC axon regeneration	van Battum et al., 2018
	Optic nerve crush (mouse)	*In vivo*	RGC/optic nerve	let7-miRNAs	Downregulation	Unclear	Induce optic nerve regeneration	Wang X.W. et al., 2018

From this review, we can see that most of the research in this field begins with bioinformatics analysis, using microarray and RNA-seq. In this way, we can discover potential regulated non-coding RNAs and predict interactive networks as well as signaling pathways. Quantitative real-time PCR (qRT-PCR) is performed to verify the expression, and lncRNAs/circRNAs-miRNA-gene regulated networks are constructed to identify target genes of certain lncRNAs/circRNAs (Yao and Yu, 2019).

Although there has been mounting evidence to show that dysregulated non-coding RNAs have a strong relationship with axon regeneration in the CNS, there is still a long way to go before we understand the precise mechanisms and methods of translational medicine.

There are some issues to be addressed in a future investigation: ① apart from SCI and optic nerve injury, there is still a lack of research into axonal regeneration-associated non-coding RNAs in other types of CNS injuries, such as traumatic brain injury (TBI). A few studies have reported that non-coding RNAs could promote neurite regeneration after TBI by attenuating inflammatory responses (Pan et al., 2017; Huang et al., 2018), but the specific mechanisms of axon regeneration are still elusive. ② Some previous studies only proved that circRNAs were differentially expressed after CNS injuries and their specific mechanism of axon regeneration remains to be further studied in the future. ③ We still know little about the upstream factors of the non-coding RNAs involved in regeneration. ④ How can we bring non-coding RNAs into clinical application? Some scientists have pointed out that fully differentiated cells can be dedifferentiated by some reprogramming transcription factors (SOX2, Lin28, KLF4, Nanog) and revert into pluripotent stem cells (iPSC). Moreover, differentiated non-neural cells can also be reprogrammed directly into neurons without going through the stem cell phase (Qian and Zhou, 2020). One previous study showed that increased expression of the miR-200 family promotes neuronal differentiation, while decreased expression of the miR-200 family promotes neuronal proliferation by targeting SOX2 and KLF4 (Pandey et al., 2015). Perhaps more investigations should be done to clarify if non-coding RNAs can be a class of bridges to connect the reprogramming process with CNS nerve regeneration. Thus, patient-derived soma cells could be reprogrammed into neurons for future drug discovery or a source of neural grafts (Faravelli and Corti, 2018). In a series of studies, extracellular vesicle localized miRNAs have been shown to improve neurological function and promote neural health, which offers a potential therapeutic strategy for our topic (Blandford et al., 2018). Furthermore, non-invasive therapeutic approaches for delivering non-coding RNAs, such as miRNA agomir/mimics or miRNA hairpin inhibitor/antagomir by oral or intravenous injection, are more available and safer. In addition, exosomes are probably also ideal vesicles as they provide a new method for the transportation of non-coding RNAs (Schorey and Bhatnagar, 2008). We still need to verify the consistency of results in animal models and human patients. In addition, much attention should be paid to safety and ethical issues when applied to patients.

In a word, non-coding RNAs provide us with further clues to understanding the underlying mechanisms of CNS nerve regeneration, and more efforts are needed to promote the application of non-coding RNAs to become therapeutic targets after CNS injuries.

## Author Contributions

PL drafted the manuscript. YJ and WT discussed the manuscript. PL, QC, and ML made the figures and tables. JJ designed the study and revised the manuscript. All authors contributed to the article and approved the submitted version.

## Conflict of Interest

The authors declare that the research was conducted in the absence of any commercial or financial relationships that could be construed as a potential conflict of interest.
